# Supplementary figures and images for: Limits to Causal Inference with State-Space Reconstruction for Infectious Disease
Source: PLoS One. 2016 Dec 28;11(12):e0169050. doi: 10.1371/journal.pone.0169050 (PMC5193453; doi:10.1371/journal.pone.0169050)

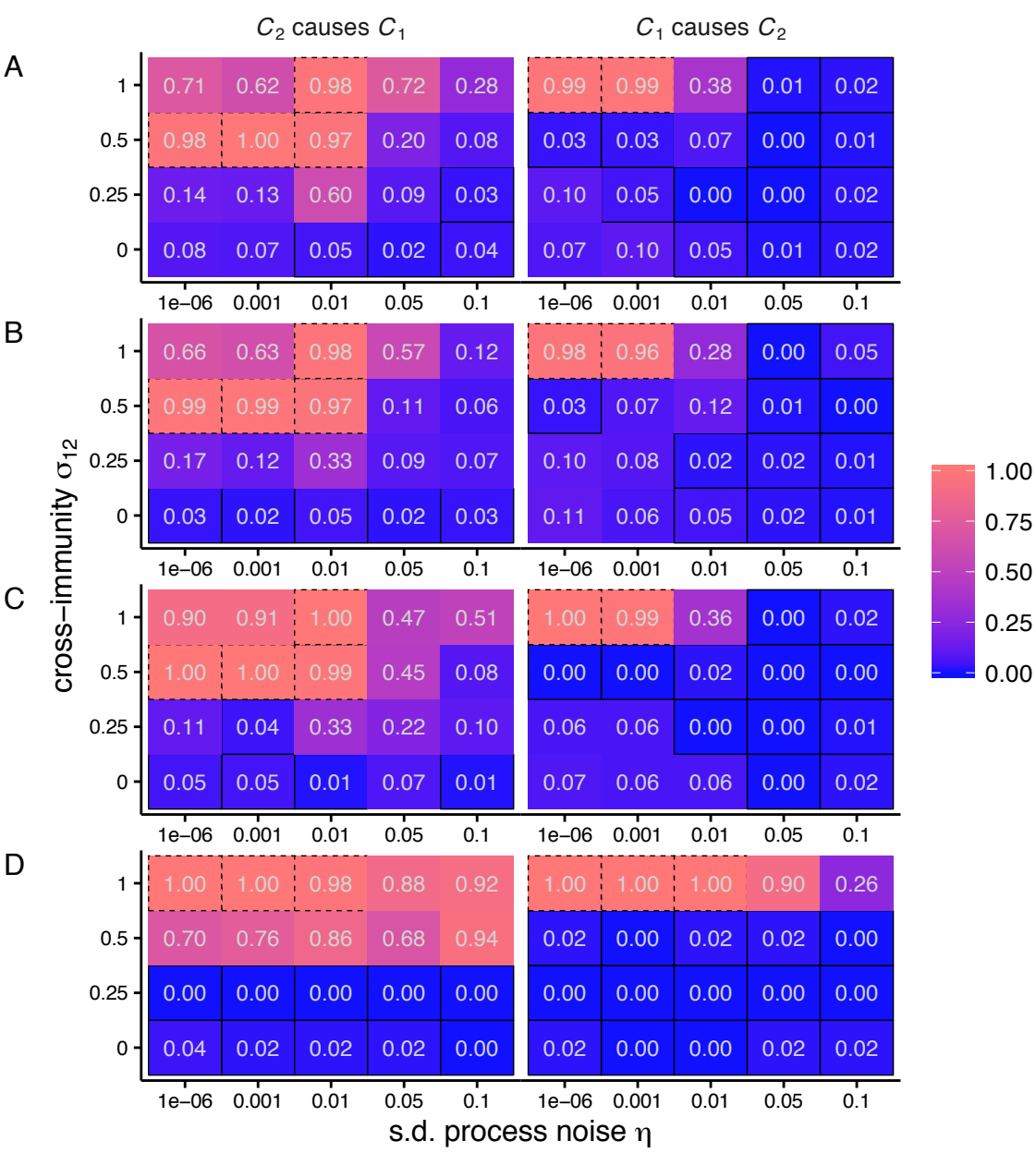

Supplement: S1 Fig — Heat maps show the fraction of 100 replicates significant for each inferred interaction for different parameter combinations. A significant increase in cross-map correlation ρ with library length L indicated a causal interaction. Each analysis is based on 1000 years of data. (A) Annual incidence, (B) prevalence strobed annually, (C) first-differenced annual incidence, and (D) monthly incidence without seasonal forcing. (PDF) [file pone.0169050.s001.pdf]

A

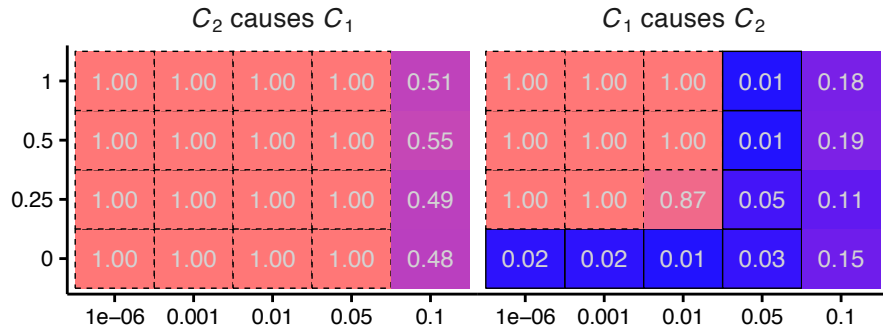

B

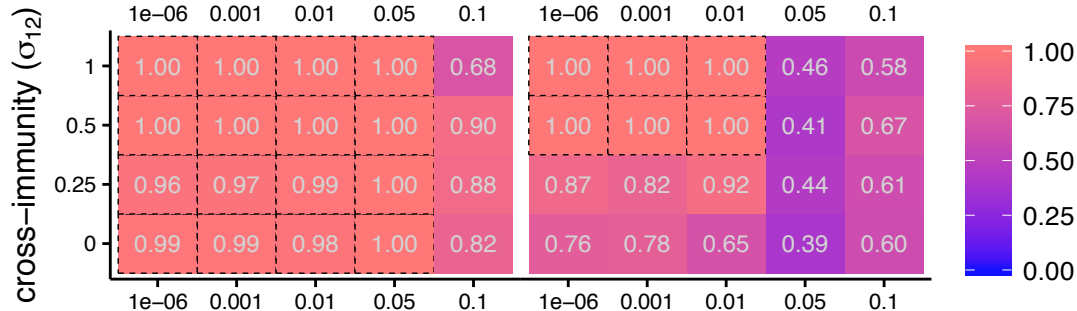

C

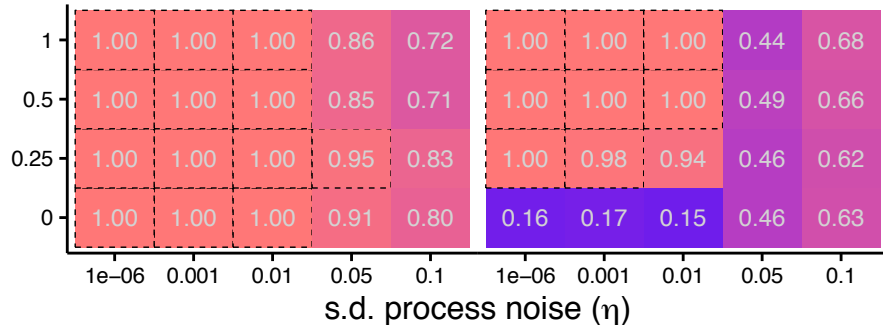

Supplement: S2 Fig — Heat maps show the fraction of 100 replicates significant for each inferred interaction for different parameter combinations. A significant increase in cross-map correlation ρ with library length L indicated a causal interaction. Each analysis is based on 100 years of monthly data. Delay-embeddings were chosen by (A) nonuniform embedding, (B) random projection, or (C) maximizing the cross-map correlation ρ. (PDF) [file pone.0169050.s002.pdf]

A

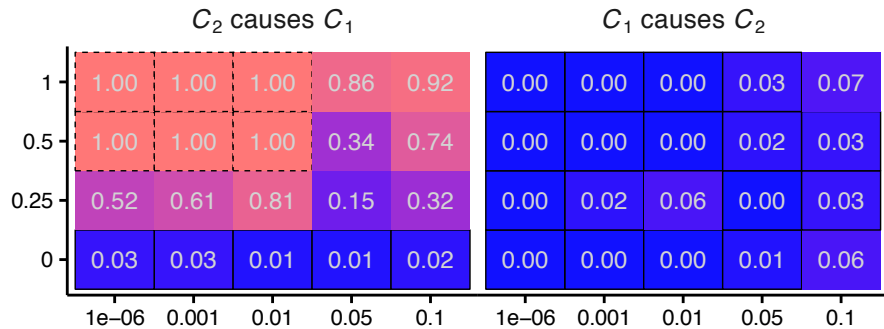

B

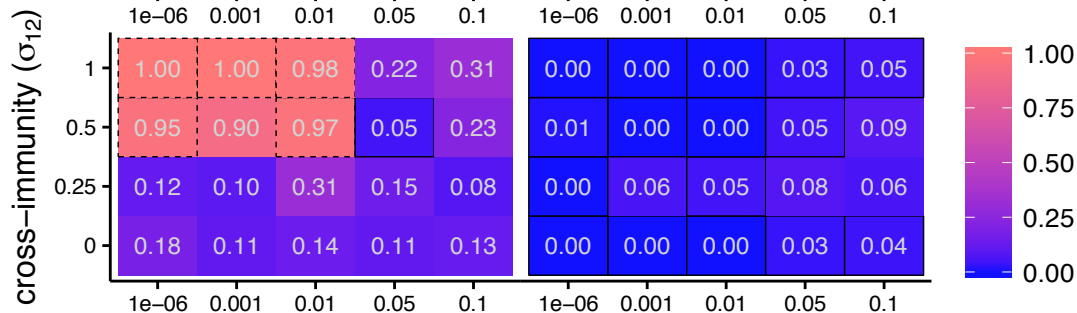

C

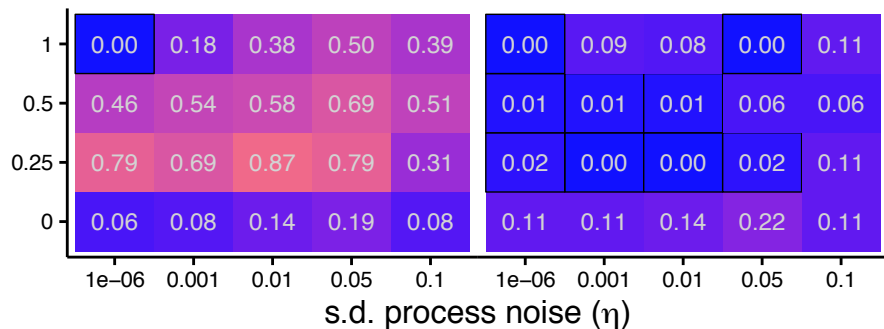

Supplement: S3 Fig — Heat maps show the fraction of 100 replicates significant for each inferred interaction for different parameter combinations. A maximum cross-map correlation ρ at a negative lag was required for inferring causal interaction. (A) 1000 years of annual incidence, requiring that the maximum ρ be positive. (B) 100 years of monthly incidence, requiring that the maximum ρ be increasing. (C) 100 years of monthly incidence with identical strains (β1 = β2 = 0.3), requiring that maximum ρ be positive. (PDF) [file pone.0169050.s003.pdf]

A

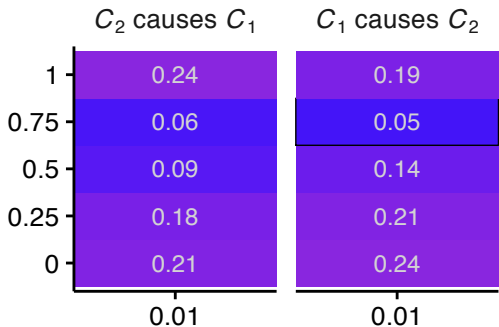

B

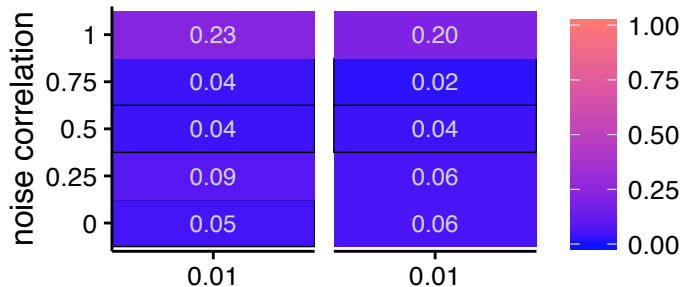

C

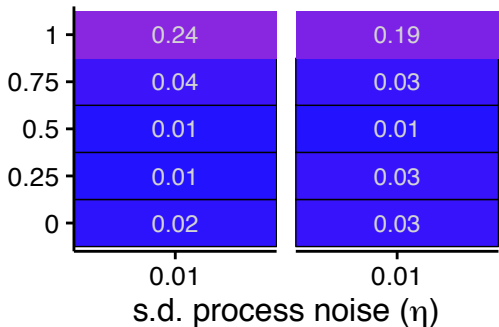

Supplement: S4 Fig — Heat maps show the fraction of 100 replicates significant for each inferred interaction. A maximum cross-map correlation ρ at a negative lag was required for inferring causal interaction. 100 years of monthly (A) and 1000 years of annual (B) incidence, requiring that the maximum ρ be positive. (C) 100 years of monthly incidence, requiring that maximum ρ be increasing. (PDF) [file pone.0169050.s004.pdf]

$C_2$  causes  $C_1$ 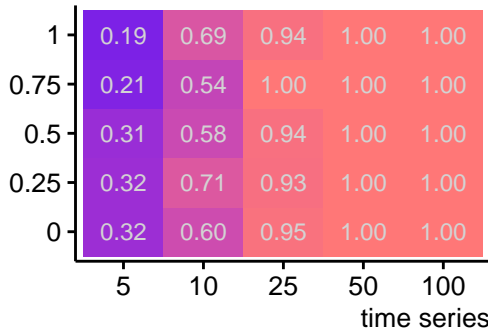 $C_1$  causes  $C_2$ 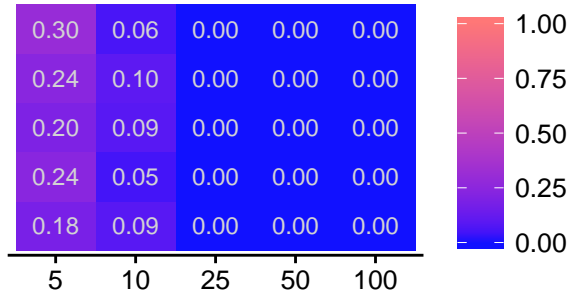

Supplement: S5 Fig — Heat maps show the fraction of 100 replicates significant for each inferred interaction. A maximum cross-map correlation ρ at a negative lag and ρ > 0 were required for inferring causal interaction. Results are shown for 5, 10, 25, 50, and 100 years of monthly incidence. (PDF) [file pone.0169050.s005.pdf]

A

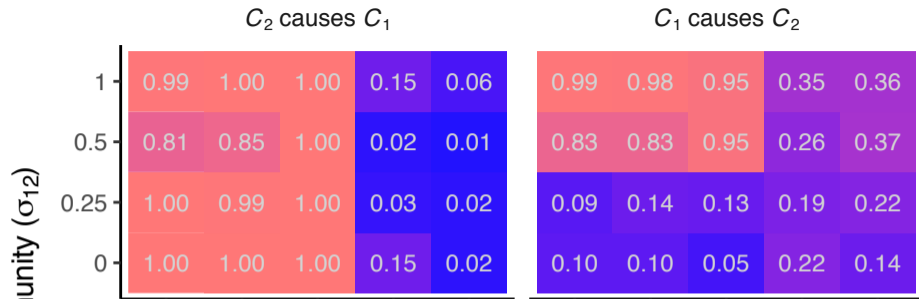

B

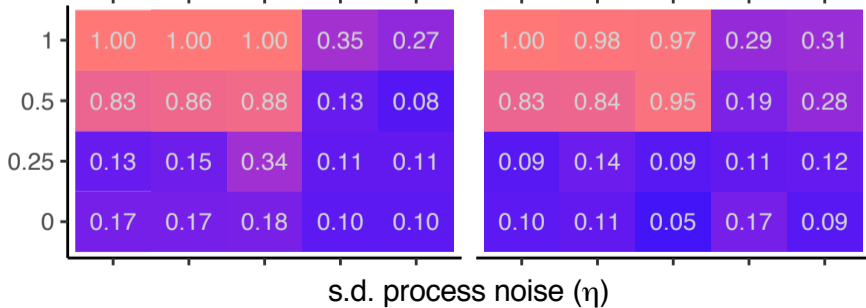

Supplement: S6 Fig — Heat maps show the fraction of 100 replicates significant for each inferred interaction in simulations using distinct strains and seasonal forcing. (A) Surrogate time series generated for putative cause and putative effect. (B) Surrogate time series generated for putative cause only. (PDF) [file pone.0169050.s006.pdf]

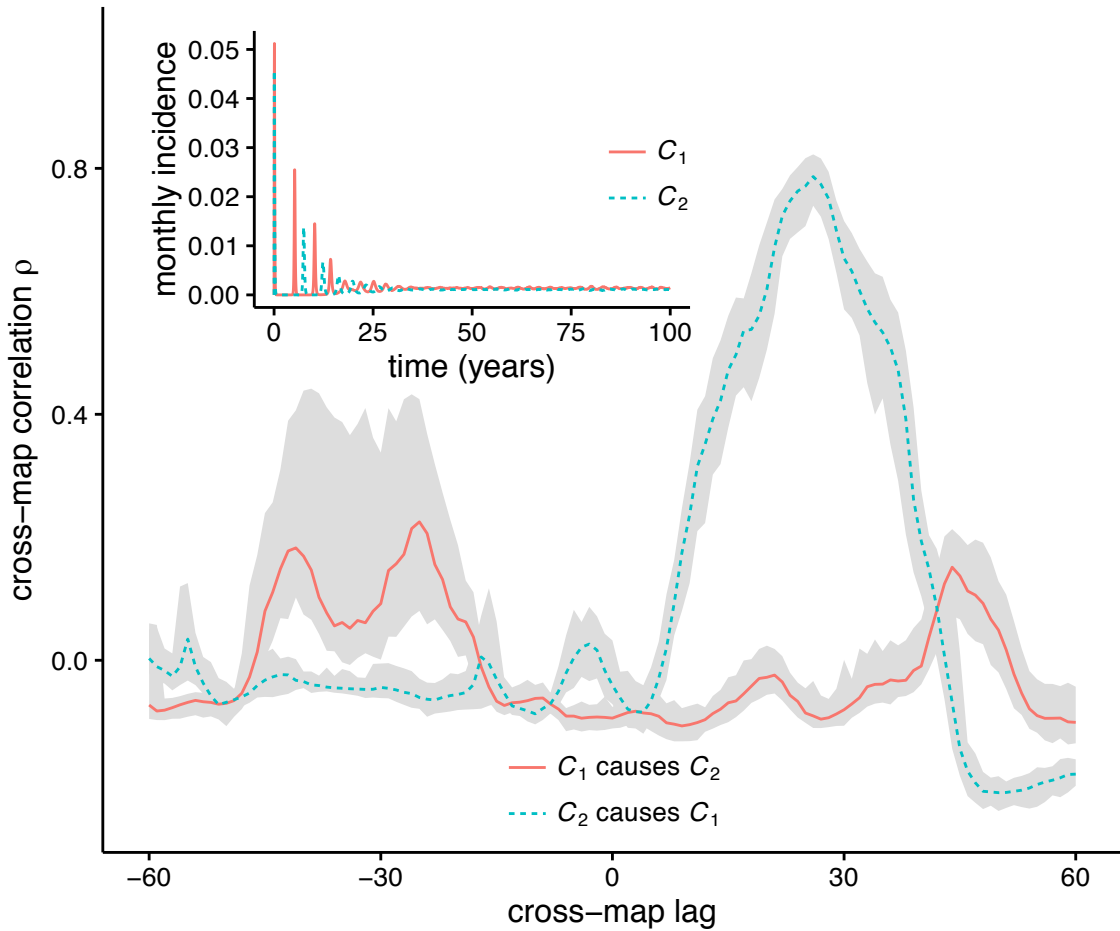

Supplement: S7 Fig — Cross-map correlations at different lags for a sample 100-year time series with monthly sampling (inset). Lines represent bootstrap medians; gray ribbons represent the middle 95% of the bootstrap distribution. Although C2 drives C1 (σ12 = 0.5, σ21 = 0), the maximum cross-correlation ρ for C1 cross-mapped to C2 occurs at a positive lag, and the reverse at a negative lag, leading to the conclusion that C1 drives C2, and C2 does not drive C1. Sample dynamics include process noise (η = 0.01) but no seasonal forcing (ϵ = 0). (PDF) [file pone.0169050.s007.pdf]

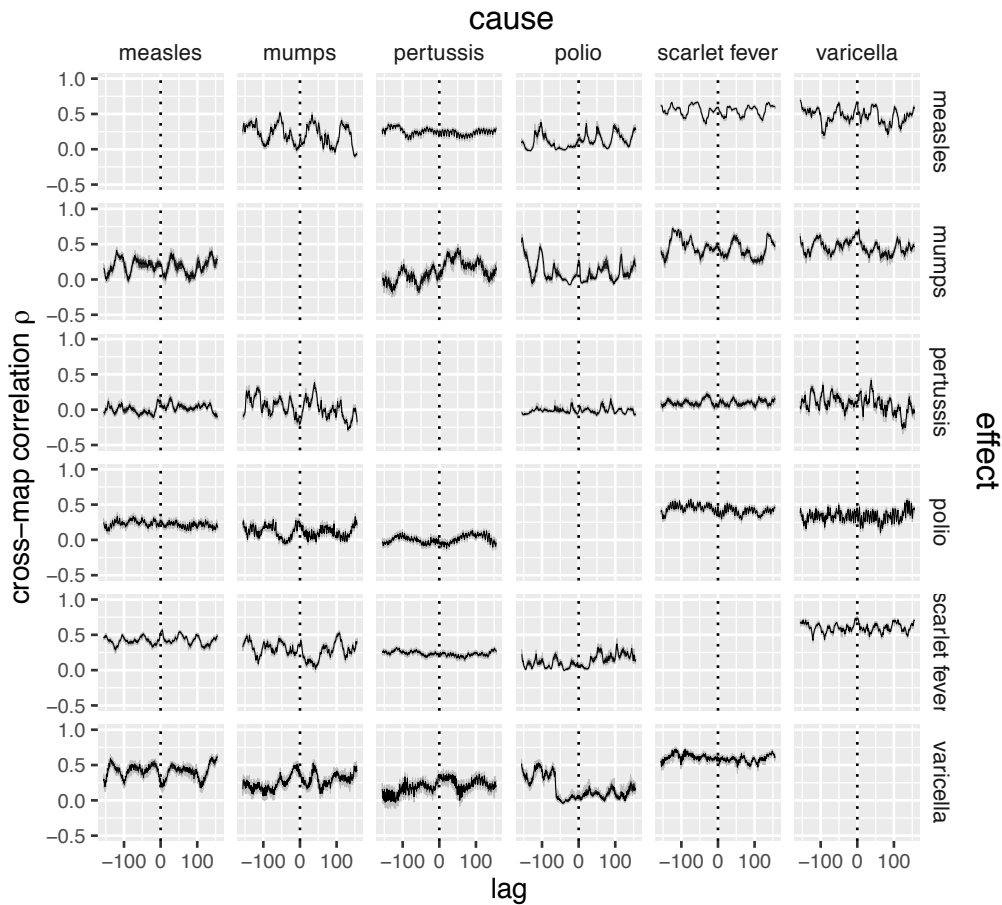

Supplement: S8 Fig — (PDF) [file pone.0169050.s008.pdf]

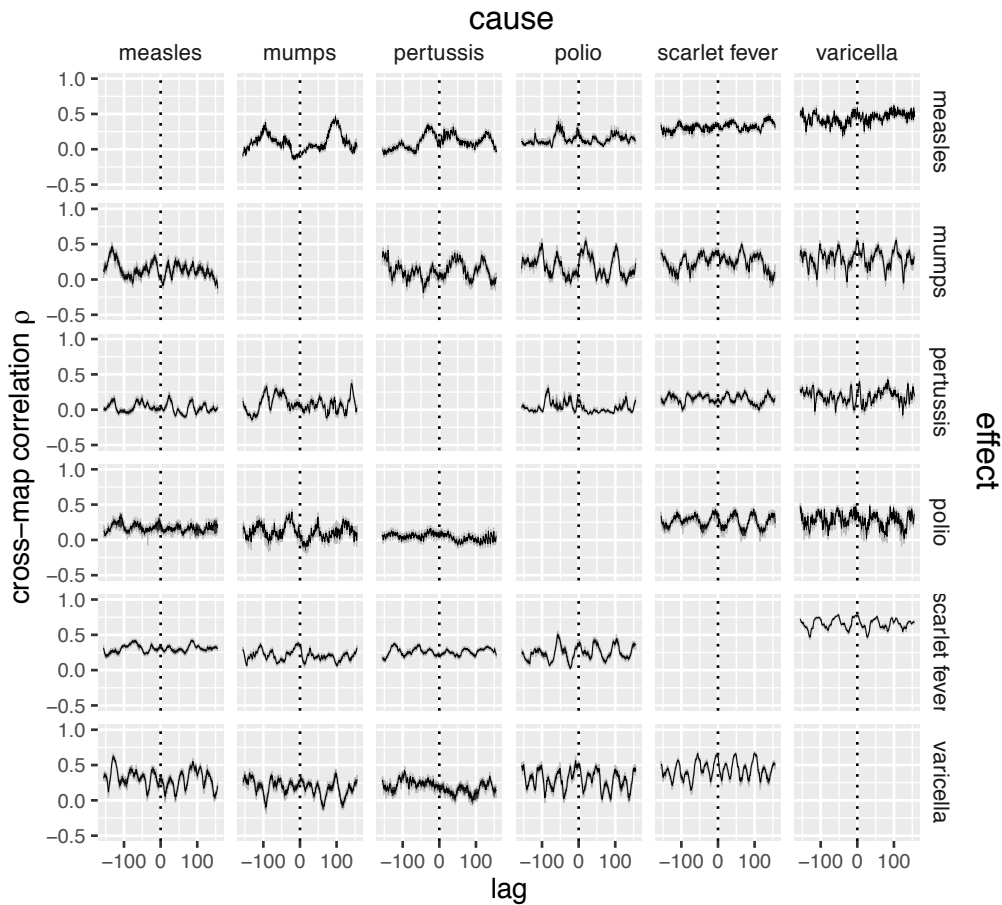

Supplement: S9 Fig — (PDF) [file pone.0169050.s009.pdf]

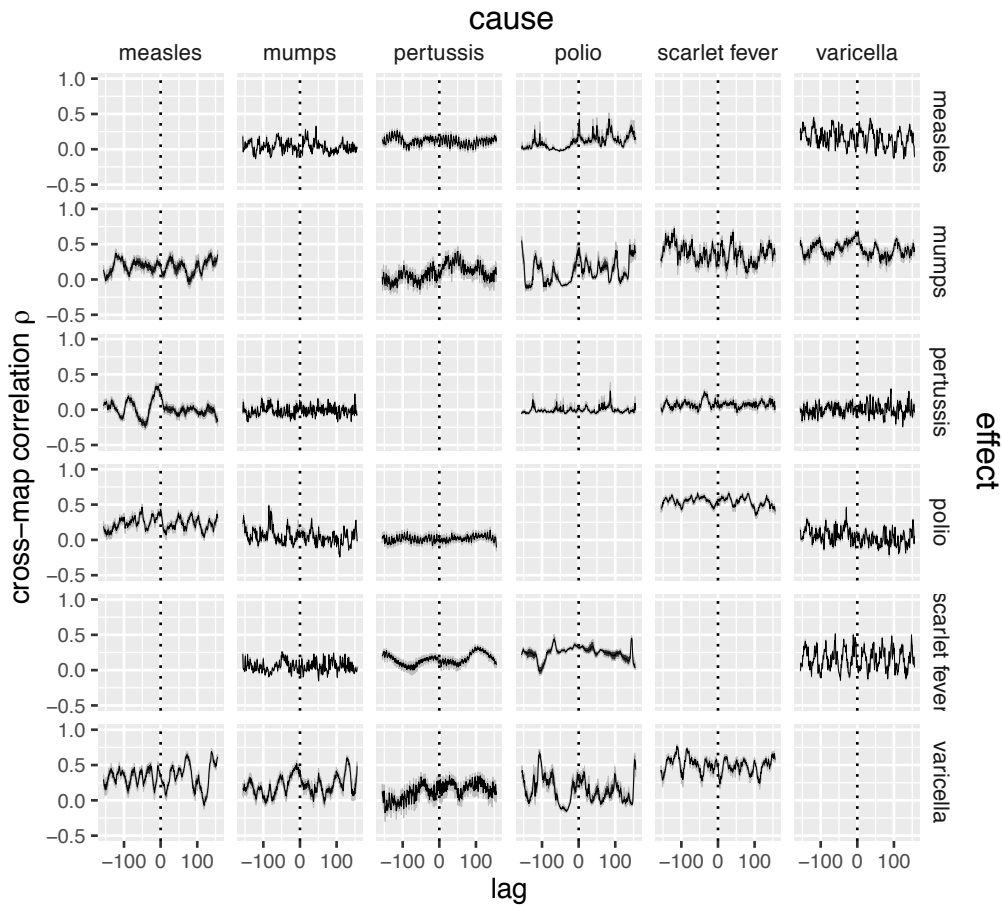

Supplement: S10 Fig — (PDF) [file pone.0169050.s010.pdf]

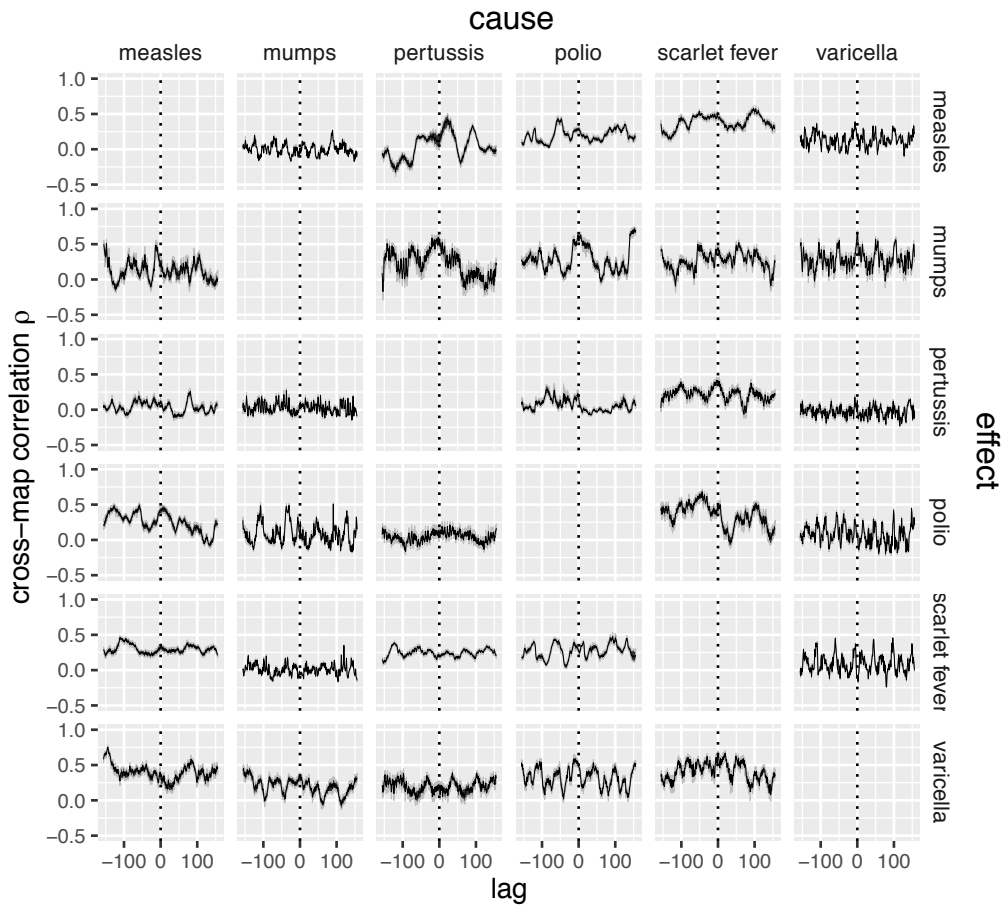

Supplement: S11 Fig — (PDF) [file pone.0169050.s011.pdf]
